# Subjective Noises in the Head and Ears: Their Etiology, Diagnosis, and Treatment

**Published:** 1891-12

**Authors:** 


*Subjective Noises in the Head and Ears: their Etiology, Diagnosis, and Treatment.* By H. MACNAUGHTON JONES, M.D., M.Ch. Pp. 152. London: BAILLIÈRE, TINDALL & COX. 1891.

The origin of this work was a paper on "The Etiology of Tinnitus," read at the Annual Meeting of the British Medical Association at Birmingham in 1890. This paper has been reproduced as Chapter I. of the present book; and other chapters on diagnosis, prognosis, and treatment have been added by the author, together with a supplementary chapter by Dr. James Cagney on the Electrical Treatment of Tinnitus, so as to make a complete work on the subject.

The result is somewhat unsatisfactory, inasmuch as the author gives us too much or too little.

In speaking of tinnitus, he roams over the whole range of ear diseases, as well as nose affections, brain lesions, &c., without being able to deal in a satisfactory way with any of them. He is evidently conscious of this; for at almost every page he refers us to another of his works, *The Practitioner's Handbook of Diseases of the Ear and Naso-pharynx*, where the subjects are more fully treated. The author dedicates the book to Dr. Adam Politzer, and in the preface he expresses obligation to this distinguished Professor for his kind perusal of the text before going to press, and gratification at his verdict that "he did not consider it necessary to add any notes to the work," which strikes us as a testimonial framed on the same lines as those published by Mark Twain from various great men of Europe on his celebrated war map. The book is written in the pleasant style characteristic of the author, and contains a short account of the latest investigations into the physiology of a most difficult subject, as well as some good clinical rules for diagnosis and treatment.

